# Sentiment and emotion in financial journalism: a corpus-based, cross-linguistic analysis of the effects of COVID

**DOI:** 10.1057/s41599-023-01725-8

**Published:** 2023-05-09

**Authors:** Chelo Vargas-Sierra, M. Ángeles Orts

**Affiliations:** 1 IULMA, University of Alicante, Alicante, Spain; 2 University of Murcia, Murcia, Spain

**Keywords:** Language and linguistics, Finance

## Abstract

Sentiment and emotion play a crucial role in financial journalism, influencing market perceptions and reactions. However, the impact of the COVID-19 crisis on the language used in financial newspapers remains underexplored. The present study addresses this gap by comparing data from specialized financial newspapers in English and Spanish, focusing on the years immediately prior to the COVID-19 crisis (2018–2019) and during the pandemic itself (2020–2021). We aim to explore how the economic upheaval of the latter period was conveyed in these publications and investigate the changes in sentiment and emotion in their language compared to the previous timeframe. To this end, we compiled comparable corpora of news items from two respected financial newspapers (*The Economist* and *Expansión*), covering both the pre-COVID and pandemic periods. Our corpus-based, contrastive EN-ES analysis of lexically polarized words and emotions allows us to describe the publications’ positioning in the two periods. We further filter lexical items using the CNN Business Fear and Greed Index, as fear and greed are the opposing emotional states most often linked to financial market unpredictability and volatility. This novel analysis is expected to provide a holistic picture of how these specialist periodicals in English and Spanish have emotionally verbalized the economic havoc of the COVID-19 period compared to their previous linguistic behaviour. By doing so, our study contributes to the understanding of sentiment and emotion in financial journalism, shedding light on how crises can reshape the linguistic landscape of the industry.

## Introduction and objectives

After years of seemingly unending prosperity, the onset of the Global Systemic Crisis in 2008 engendered a new discourse on economic meltdown, bringing into public prominence the language of finance and economics in the broadcast and print media, with these outlets effectively coming to act as spokespeople for banks, financial institutions and even governments in the ensuing years (Kelsey et al., 2018). Newspaper articles and financial reports are key sources of information for investors in making decisions on investments, forming financial policies, and so on (Shalini, 2014, p. 270). But more recently two factors, the worsening of climate change (Nordhaus, 2019) and, more significantly, the economic effects of the COVID-19 pandemic^1^, led to a worsening economic outlook, this at a time when the world economy was still recovering from the throes of the great recession that began in 2008. The pandemic resulted in unforeseeable economic mayhem, a perfect storm arising from supply-chain shortages, heightened inflation, sky-high shipping rates and climate change transition costs, a situation which has further deteriorated following the outbreak of the Ukrainian war, which continues at the time of writing.

Such events have an impact on the language used in news journalism, and linguists can seek to identify certain patterns here. Compiling and analysing a textual collection—a corpus—according to those parameters that best fit the purpose of linguistic research has proved to be an effective way of studying language in use, since a corpus-based approach provides a sound methodology, one that draws on systematic observation and experimentation, as well as making the verification of results possible.

The present study focuses on two comparable corpora, one in English (almost four million words) and one in Spanish (almost two million words), each comprising two sub-corpora from the immediate pre-COVID period (2018–2019) and the main years of the pandemic itself (2020–2022). These are compiled of news items from two prestigious financial periodicals, *The Economist* and *Expansión*, and thus represent the situation a decade after the 2008 crisis and during the COVID crisis. Our premise is that emotions play a key role in economic behaviour and decision-making (Berezin, 2005, 2009; Seki et al., 2021, among others). Accordingly, our main research objective is to illustrate and measure business sentiment and emotions on the basis of linguistic data from newspaper articles published during the two periods under analysis. Against the background of an enduring and complex economic crisis, one in which the first signs of recovery were being seen after 10 years of slow economic activity, we expect that the lexicon and general tone of the news items in the first period will be mildly positive in both corpora, yet still coloured to some extent by negative sentiments, this reflecting the long-lasting effects of the prior economic depression. We also predict that a dramatic worsening of tone will be perceived in the second period of analysis for both corpora, since at this time many adverse contingencies are at play, especially the pandemic, but also the deteriorating state of the climate crisis. We also hypothesize that the polarization of sentiment in each period and variations in expressions of emotion might differ depending on the periodical, activating either fear or greed as the underlying factor in the unpredictability and volatility of financial markets.

## Theoretical framework

### A history of two crises

The following lexical study can be seen within the following contextualisation. In the immediate pre-COVID period (2018–2019), the effects of the 2008 meltdown were still making themselves felt: even after a decade, recovery in some sectors remained sluggish. Exceptional and indeed unprecedented policies had been adopted by most countries in response to the recession, but momentum had been slow-moving and subdued macroeconomically (Chen et al., 2019), this is due to illiquidity, credit impairment and capital shortfalls, as noted by the IMF World Economic Outlook^2^. The policies of major central banks in this period were vital for recovery, in that until very recently interest rates were kept low to foster liquidity. Nevertheless, there has been major tension in trade between the large economies, as well as increased trade barriers and production stoppages, most notably in the automotive industry, together with growing concerns about climate change.

In the COVID period, the ferocity of the pandemic led to a variety of lockdown measures, and in the aftermath of these came new economic challenges, including shortages of labour and parts (notably semi-conductors), shipping/transport bottlenecks, shortages in the global food supply, and crises in the services sector (travel, tourism, hospitality industry). All of the above paved the way for stagflation, that is, rising inflation and negative economic growth, and with it increases in shipping rates, rising global food prices, fuel prices at record levels, generally spiralling prices, the hoarding of resources, and the redirection of service demands towards goods, at the same time as a new fiscal orthodoxy on the part of major central banks began to tighten banking rates, with measures for economic stimulus beginning to be withdrawn in many areas. At the same time, according to the IMF’s reports, climate change has become a pressing issue, as governments have started to calculate transition costs and risks, at a time when they are also submitting financial institutions to severe stress tests in preparation for a new, more sustainable future^3^.

### Economic discourse and sentiment and emotion analysis

Emotion and sentiment are essential elements in people’s lives and are expressed linguistically through various forms of communication, not least in written texts of all kinds (news, reports, letters, blogs, forums, tweets, micro-bloggings, etc.). Sentiment is defined by Taboada (2016, p. 326) as “the expression of subjectivity as either a positive or negative opinion”. As such, sentiment analysis (SA), one of the tools to be used in our study, approaches the issue in terms of polarity in texts; that is, it seeks to establish whether an author conveys a negative, positive or neutral attitude when discussing or describing a specific object, be it a service, product, institution, company, place, etc. This type of analysis can be complemented by the detection of emotion. An emotion is a particular feeling that characterizes a state of mind, such as fear, joy, anger, or love; thus, emotion analysis determines exactly which emotional or mental states are evident in a text by identifying terms or phrases referring to the affective and emotional sphere involved (Nandwani and Verma, 2021; López-Rodríguez, 2022).

As we noted in the Introduction, this paper seeks to link sentiment and emotion with the discourse of economics and to do so both implicitly and explicitly. In the area of linguistics, however, the connection between emotions and economic language has seldom been addressed, albeit with some recent exceptions (Devitt and Ahmad, 2007, 2010; Kelly and Ahmad, 2018; Orts, 2020a, b). Not without some justification, economics has traditionally been seen as a rational and impartial discipline, devoid of emotions and feelings (Bandelj, 2009). Nevertheless, this does not necessarily hold in other fields. Because emotions are an important feature of human nature, they have attracted a great deal of attention in psychology and other fields of study relating to human behaviour, like business, healthcare, and education (Nandwani and Verma, 2021). For example, in computer science and computational linguistics, the question of how to automatize sentiment and the detection and analysis of emotion have both been the focus of a great deal of research, especially in terms of human–computer interaction (Strapparava and Mihalcea, 2008) and opinion mining (Taboada, 2016).

Incidentally, rational choice theory in economics, as the best-established theory on investment behaviour, considers that individuals react predictably and rationally in terms of economic or financial decisions (Zey, 1998).

However, studies on emotion in the fields of philosophy and psychology have shown that emotions should be understood as complex mental states, whose existence is not separable from other mental states, and which are essential to understanding human thought and activity (Deigh, 2008, p. 3; Lewandoska-Tomaszczyk et al., 2020). In the same vein, Damásio (2018) and TenHouten (2014) also refute the existence of the reason–emotion duality, arguing that emotions are fundamental in decision-making and goal-formation. Specifically in the field of economics, economists and sociologists (Montier, 2002; Berezin, 2005, 2009) highlight the powerful role of emotions in economic performance, where many important decisions, such as large-scale purchases or sudden withdrawals of investments, are made by individuals while experiencing impulsive moods. Not surprisingly, “greed and fear are two concepts widely used in experimental financial economics” (Barone-Adesi et al., 2018, p. 46) and constitute two divergent emotional states that underlie market uncertainties and volatilities.

A key indicator in economic decisions is the Fear & Greed Index, developed by CNN^4^ and pioneered by the financial behaviourist Montier (2002), who postulates that financial sentiment is driven by two opposing forces: greed, when the market is buoyant (shares attain prices above their real value in a bull market) and fear of loss, when markets slow down (shares are priced below their real value in a bear market) (Cipollini and Manzini, 2007). In financial terms, fear is gauged by the level of volatility (which implies uncertainty or risk aversion), which in turn is measured by the Chicago Board’s VIX or Volatility Index: the lower the volatility, the higher the level of greed. Auinger (2015, p. 39) has argued that, irrespective of how sophisticated the mathematical equations to calculate sentiment are, no statistically causal relationships exist between different measures of confidence or insecurity. Emotions, on the other hand, play a significant role here and have a powerful influence on market decisions, something which can be traced historically.

According to Plamper and Lazier (2001, pp. 134–135), the decade between 1990 and 2000 was an era of optimism on the part of investors, but this dissipated with the bursting of the dot.com bubble, and confidence only began to build again from 2003 onwards. Greed reached a new peak in 2007, on the eve of the Global Systemic Crisis, the worst downturn since the Great Depression, which caused a massive sell-off or *contagion*, one which, we postulate, has persisted into both of the two-year periods we are analysing here. This study, indeed, seeks to illustrate how fear and greed as expressions of emotions occur verbally in the news of the two periods considered.

Plutchik’s model of eight primary emotions (2001; Plutchik and Kellerman, 2013) is ultimately inspired by Darwin’s theory of evolution and states that existential life problems (identity, temporality, territoriality, and hierarchy) are associated with positive and negative responses in the form of primary emotions: trust/disgust, joy/sadness, anger/fear and anticipation/surprise, as shown in Table 1, which is based on TenHouten’s interpretation (2014, p. 17).

**Table 1:** Plutchik’s four existential problems and eight primary emotions (after TenHouten, 2014, p. 17).

Existential problems	Primary emotion	Stimulus and cognitive process
Hierarchy (moving toward obstacle/moving away from threat)	anger	Pursuit approach/avoidance of risks
	fear	
Temporality (possession; deprivation)	joy	Retaining, possessing/abandoning
	sadness	
Identity ((self-) acceptance; rejection of others)	trust	Taking in/rejecting
	disgust	
Territoriality (exploration; opening or closing boundaries)	anticipation	Examine, study
	surprise	

**Fig. 1:**
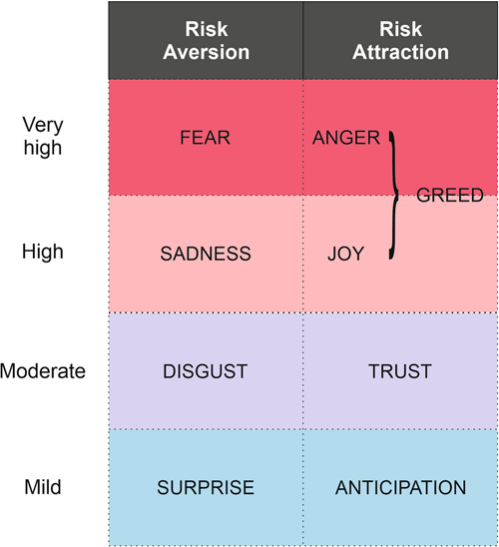
Plutchik’s taxonomy adapted to the Fear and Greed Index.

Plutchik argues that the eight basic emotions form four opposing pairs, i.e., joy–sadness, anger–fear, trust–disgust, and anticipation–surprise. By adapting Plutchik’s taxonomy (Fig. 1), we can fit the emotions to the Fear and Greed Index, and thus grade them in terms of their relation to being more or less prone to risk aversion or risk attraction.

For the purposes of our study, and within the context of the financial markets, the eight emotions exist in a range from very high to mild emotional intensity, where the extremes are fear (the terror of facing a threat), and its opposite, greed, which we conceive as a far more complex sentiment than fear, and one which is not considered a primary emotion by Plutchik.

Because fear constitutes a dulling of the self, a cowardly shying away from obstacles (TenHouten, 2014, p. 134), it is simpler to define, belonging, as it does, to Plutchik’s wheel of emotions and having been amply discussed and described. Affect Spectrum Theory (AST), which belongs to the terrain of neurosociology (TenHouten, 2014), does not mention greed nor does it discuss it. In fact, greed is a key, and complex, construct that has not really been entirely deciphered by Behavioural Finance.

From the point of view of Psychology, greed has been defined as: “the desire to acquire more, and the dissatisfaction of never having enough” (Seuntjens et al., 2015, p. 519), which invests it with a negative hue. However, a precise definition of the concept is the subject of much undefined academic discussion (Razen and Stephan, 2029, p. 164), as it encompasses a range of motivations and desires related to acquiring wealth and possessions, which makes subjects eager to overcome obstacles or challenges in their pursuit of wealth or success (Sivanathan and Pettit, 2010; Keltner et al., 2003).

Thus, for the purposes of our study, we needed to integrate greed into the AST paradigm, without losing sight of market theory. In order to make the study feasible using Plutchik’s taxonomy and Tenhouten’s interpretation, we decided to devise greed as a secondary emotion combining two primary emotions: happiness (joy, which Plutchik and TenHouten define as the need to possess and gain social support, in TenHouten, 2014, p. 17) +zest (anger, the appetite to move forward and overcome obstacles) as the key sentiments that combine to spark utter optimism in financial decision-making. In other words, fear in CNN’s scalar range represents aversion to invest, while within the scope of our study, the sum of anger and joy equates to greed, and hence represents the audacity to invest, no matter the obstacles, given that these can be overcome. In addition, anger and joy are functionally aimed at personal well-being: anger arises from the fierce ongoing fight for one’s resources (Panksepp, 1988, in TenHouten, 2014, p. 139), while joy is the result of rewarded progress toward those goals.

Following our current model, greed would arise from the stamina to succeed in attaining and possessing, whereas fear and sadness (as the opposite of joy) are both avoidance-oriented and intuitive, a “recoiling and reaction to dangerous and undesirable situations” (TenHouten, 2014, p. 152), with the emphasis on risk aversion and escape from threat.

What we call *moderate* emotions, disgust and trust, are two sides of the same coin, and are marginally less intense in terms of risk aversion/attraction, since they are oriented towards expelling or incorporating something as adaptive responses to negative or positive experiences (TenHouten, 2014, p. 165). Surprise and anticipation are also aimed at the achievement of goals but are considered mild emotions regarding aversion/disposition to invest. In our corpus, in both English and Spanish, surprise, depending on the words involved, is tagged as positive (‘money’, ‘deal’, ‘hope’ or ‘shopping’) or negative (‘disruption’, ‘emergency’, ‘epidemic’, ‘disaster’, ‘shock’), almost in equal proportions. When Surprise is negative, it is because of a thwarting of expectations due to a mismatch between what is predictable, or new and unfavourable information, like in the following examples taken from *The Economist*: (1) “On top of uncertainty in Iran, disruption in Venezuela, Libya, Nigeria or Iraq could squeeze global supply”; (2) “But a shock could put corporate America into trouble”; or (3) “These speculative positions are vulnerable to a shock, such as a sudden rise in interest rates, which can turn into a fully-fledged crisis”; anticipation, on the other hand, is definitely positive, both in English and Spanish according to the data in our corpus (208 positive nouns in English versus 54 negative; 93 positive verbs in English versus 24 negative; 103 positive nouns in Spanish versus 15 negative, for example) or it was tagged without polarity (neither positive nor negative, 88 nouns in English, 46 in Spanish); when tagged positively in words, it constitutes the capacity to (successfully) infer something from previously acquired information.

In sum, an increase in fear, sadness, disgust, or surprise will reveal themselves, to a greater or lesser extent, in a risk-aversion response to investing, while an increase in anger/joy, trust and anticipation will be (gradual) indicators of a willingness to invest. We will first analyse the occurrence of these indicators in our general financial corpora, before focusing more closely on the main emotions that move financial markets: fear and greed.

## Corpus description and methodology

For the present study, we adopted a corpus-based methodology, which involved compiling a representative sample of the material under examination, plus the use of a series of electronic tools to extract quantitative and qualitative data. The identification of sentiment and emotion in text corpora is an increasingly productive line of research, in that it allows us to understand in more detail the mood, opinion, subjectivity, and point of view expressed about certain products, services, places, governments, and public or private organizations; as such it helps us to better interpret the human experience here by means of text analysis (Taboada, 2016). In financial texts, classifying sentiment and emotion is also important in helping to predict a financial crisis and to identify stock market trends, since judging whether a financial text is positive or negative, and what predominant emotions it harbours, can provide key information on the current economic situation and on the levels of enthusiasm or pessimism with regard to investing in the markets, as noted above. After this process of classification, the data are analysed statistically to arrive at a finer-tuned assessment of the presence of emotionally charged words and phrases in the corpus texts. We used automated analysis to examine sentiment polarity and the emotions found in our two comparable ad hoc corpora of financial journalism to determine the intensity of sentiment and emotional tendencies therein.

The composition of the corpora and the tools used for the analysis are described in what follows.

## Corpora and sub-corpora

A corpus was built in English (3,736,103 words) and another in Spanish (1,965,154 words), both based on news items that appeared in two prestigious financial economic newspapers: *The Economist* and *Expansión*. The former is a world-renowned newspaper based in London which originally targeted a British readership, but today has a global, well-educated, and literate middle-to-upper class readership; while its content is basically economic and business related, it also covers political affairs, world news and a miscellanea of general news, plus art-related and literary items. The latter is a respected national Spanish newspaper with significant financial content, including translated extracts from the English *Financial Time*s. It is aimed at a Spanish-speaking, semi-specialized middle-to-upper class readership and is one of the most-read publications of its kind.

For the purposes of finding and downloading appropriate texts, we consulted two databases, both accessible through the library of the University of Alicante: ProQuest Central, to access *The Economist*, and Dow Jones Factiva, for *Expansión*. We sought all news items dealing with climate change, the economic crisis, interest rates, and the COVID pandemic. Consequently, the search terms in the two languages were the same, although specific to each period. For the period 2018–2019 the search terms were: “climate change” AND “economic crisis” OR “havoc” OR “interest rates”; for 2020 and 2021 years the words were: “covid” AND “climate change” AND “economic crisis” OR “havoc” OR “interest rates” OR “pandemic”. For the overall *Expansión* corpus, the resulting sample size was smaller (a total of 1589 downloaded files) than the overall data set for *The Economist* (3735 files), hence a difference of some 2146 news items across the two periods, representing a difference of about one and a half million words (Table 2).

**Table 2:** Corpora statistics.

	EN files	The Economist (words)	ES files	Expansión (words)
2018	384	433,422	287	221,378
2019	434	452,255	576	417,904
2020	1463	1,452,191	216	550,064
2021	1454	1,398,235	510	775,808
Total	3735	3,736,103	1589	1,965,154

Once the general financial corpora had been compiled, two sub-corpora were made for each language and newspaper, which we called pre-COVID, containing the texts from 2018 to 2019, and COVID, comprising material from 2020 to 2021. Table 3 sets out the total number of tokens and words^5^ for each of these, together with percentages for the overall corpus.

**Table 3:** Sub-corpora statistics.

Sub-corpora	Tokens	Words	%
Pre-COVID *The Economist*	1,029,509	~886,168	23.7
COVID *The Economist*	3,310,942	~2,849,935	76.3
Pre-COVID *Expansión*	740,395	~632,130	32.2
COVID *Expansión*	1,561,339	~1,333,024	67.8

### Lingmotif 2 software: positive and negative polarities

With the aim of measuring sentiment, we conducted a preliminary analysis of sentiment in the two smaller (pre-COVID) corpora, which comprised fewer than one million words in each language (cf. Table 4). This lower number of words was necessary due to the limitations of the Lingmotif 2 software^6^ (Moreno-Ortiz, 2021). Its basic function is to determine the semantic orientation of a text, that is, the extent to which it can be said to be positive or negative, by detecting the positivity or negativity contained in the different linguistic expressions in the text(s) analysed. It differs from some other opinion-mining tools because the system supports the processing of longer texts, not just mini-texts such as tweets. Additionally, it takes a dictionary-based approach, i.e., it draws on an internal lexicon created to include words and phrases with an affective charge, so that these are detected in the text under analysis by comparing the input lexical units and those contained in the application’s own dictionary.

**Table 4:** Topic areas and main polarity items in the four sub-corpora.

	PRE-COVID corpus		COVID corpus	
	Expansión	The Economist	Expansión	The Economist
Lexical fields, topics and examples	**Lexical field I**			
	**Climate change**	**Economy**	**Health crisis**	**Economy**
	combustibles fósiles, efecto invernadero, transición energética, emisiones de gases, dióxido de carbono, emisiones de carbono, energía solar, ministra de transición, huella de carbono	financial crisis, climate change, monetary policy, percent of GDP, trade war, rich countries/world, economic growth, world/global economy single market, foreign investors, unemployment rate, government bonds, fiscal stimulus, private sector, budget/current account deficit, tax cuts, share/oil prices, labour market, government/public debt	nuevos casos, estado de alarma, toque de queda, tasa de positividad, casos de coronavirus, nuevas infecciones, unidades de cuidados, nuevas medidas, consejería de sanidad, prórroga del estado, nueva normalidad	interest rates, percent of GDP, central banks, rich countries, financial crisis, climate change, rich countries, supply chains, global financial crisis., public debt, financial markets, fiscal stimulus, low interest rates, private equity, private /public markets, Chinese banks, venture capital, market share, economic crisis, monetary policy, share of GDP, global supply chains, inflation expectations, tech giants
	**Lexical field II**			
	**Business innovation**	**Banks**	**Political affairs**	**Health crisis**
	puesta en marcha, nuevo plan, efectos del cambio, creación de valor, carriles bici, nuevas líneas	interest rates, central bank, monetary policy, low interest rates, bank of England, big banks, quantitative easing	recuento de votos, próximo presidente, nuevo presidente, servicio secreto, gobierno regional, campaña de reelección, jefa de campaña, candidato ganador, gobierno francés	health care, second wave, public health, immune system, death toll, pre-pandemic levels, excess deaths, medical supplies,
	**Lexical field III**			
	**Economy**	**Political affairs**	**Miscellanea**	**Political affairs**
	buen gobierno, tipos de interés, pequeñas empresas, tasa de paro, capital riesgo	prime minister, chief executive, euro area, second world war, armed forces	cinturón de oxido, energías fósiles	second world war, African countries, British government, euro zone, nuclear power, general election, national governments
Top 10 negative items	cambio climático (517), riesgo (205), imponer (148), problema (129), afectar (128), crisis (91), comprometer (84), deuda (77), amenazar (73), provocar (63)	debt (206), inflation (197), problem (149), crisis (123), risk (120), deficit (107), fear (100), lose (98), financial crisis (78), recession (76)	pandemia (887), contagio (609), fallecido (451), muerte (302), restricción (286), estado de alarma (253), incidencia (245), crisis (236), riesgo (187), toque de queda (176)	pandemic (940), virus (350), lockdown/s (350), risk (286), crisis (273), debt (267), problem (239), inflation (206), death (201), worry (192)
top 10 positive items	sostenible (244), mejorar (176), contra el cambio climático (173), garantizar (118), aprobar (110), innovación (106), confianza (99), solución (88), mejor (86), apostar por (84)	help (168), support (121), rich (111), wealth (96), win (88), profits (85), good (84), benefit (78), best (68), free (68)	positivo (633)^a^, ganar (595), victoria (402), ventaja (289), ganador (265), hospitalario (188), positividad (169), aprobar 8111), aventajar (104), favorito (103).	help (410), rich (264), support (257), benefit (187), good (160), win (146), best (126), great (99), success (89), protect (87)

^a^The word *positivo* was tagged as a positive item by Lingmotif 2, but in our COVID corpus it will undoubtedly have a negative polarity in most cases, in light of its specific virus-related context of use. Predicting the correct polarity is often challenging for lexicon-based software, which use dictionaries of words tagged with their respective polarity. The contexts in which *positivo* appeared in our COVID corpora (e.g., *caso positivo*, or ‘positive case’) becomes decisive in determining its polarity or emotion.

Lingmotif 2 (Moreno-Ortiz, 2021) uses a scale from 0 to 100 to categorize texts, from ‘extremely negative’ to ‘extremely positive’, based on the semantic orientation of the sentiment detected in the text (Text Sentiment Score, or TSS). The TSS calculates the polarity of each sentence, taking into account both the number and the position of sentiment-related items. Following this, the Text Sentiment Intensity (TSI) is calculated by weighing the number of positive and negative sentences. Apart from assigning values from −5 to +5 to the sentiment items (with 0 indicating an expression of neutrality) the software also uses context rules (of inversion, intensification, and attenuation) to accommodate possible sentiment modifiers (Moreno-Ortiz, 2017, p. 133).

As noted above, in order to work with the most recent version of Lingmotif, a reduced sample of less than one million words per language was required. To obtain this scaled-down sample we considered the quantitative proportion of words (%) in each year for both corpora and thus arrived at the number of words we needed, as shown in Table 5. Files were randomly selected for each year until the approximate number of words we required was reached.

**Table 5:** Sample corpora for SA using Lingmotif 2.

Year	English			Spanish		
	Words	%	Sample corpus	Words	%	Sample corpus
2018	433,422	11.6	118,996	221,378	11.27	111,317
2019	452,255	12.1	124,068	417,904	21.27	210,251
2020	1,452,191	38.87	378,996	550,064	27.99	276,877
2021	1,398,235	37.42	378,009	775,808	39.48	403,414
Total	3,736,103		1,000,069	1,965,154		1,001,859

Lexical items were filtered through CNN’s Fear and Greed Index, in that these are the two opposing emotional states that cause unpredictability and volatility in financial markets. To identify this kind of inscribed or indirect meaning, Plutchik’s model was used along these two scalar polarities: from the negative (fear) to the positive (greed) with the aid of EmoLex, a word-emotion lexicon which will be described below. Through conducting the study in layers, our goal was to provide a holistic explanation of how English and Spanish periodicals had emotionally verbalized the economic havoc taking place immediately before and during the COVID pandemic.

### Applying the NRC word–emotion association Lexicon

**Fig. 2:**
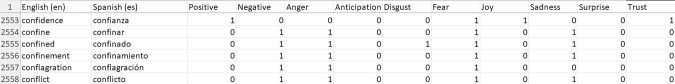
EmoLex in English and Spanish.

For this part of our study, we used pre-covid expansión, pre-covid economist, covid expansión and covid economist (see Table 3). With each sub-corpus, we worked with word frequency lists extracted using Sketch Engine (Kilgarriff et al., 2004) and then checked these words against the NRC Word–Emotion Association Lexicon, also known as EmoLex (Mohammad and Turney 2013; Mohammad, 2018, 2020), itself based on Plutchik’s eight basic emotions. EmoLex was used to assign scores for positive/negative sentiment and for potential context-invariant emotions. This lexicon is a list of more than 14,000 English unigrams and their equivalents in other languages, including Spanish^7^. These unigrams are associated with eight primary emotions: anger, fear, anticipation, trust, surprise, sadness, joy, and disgust, corresponding to Plutchik’s taxonomy (2001 version). The NRC sentiment lexicon makes a binary categorization of words into positive and/or negative and into one or more emotions since they are not mutually exclusive (Fig. 2):

We cross-checked the word frequency lists obtained with Sketch Engine (SkE) with the EmoLex list by language. The assignment of emotions to a word in our frequency lists was done automatically using advanced Excel conditional search formulas. The condition was set up as follows:

$$= {\rm {XLOOKUP}}\left( \begin{array}{l}lookup\_value,\,lookup\_array,\,return\_array,\\ \left[ {if\_not\_found} \right],\,\left[ {match\_mode} \right],\,\left[ {search\_mode} \right]\end{array} \right)$$

**Fig. 3:**
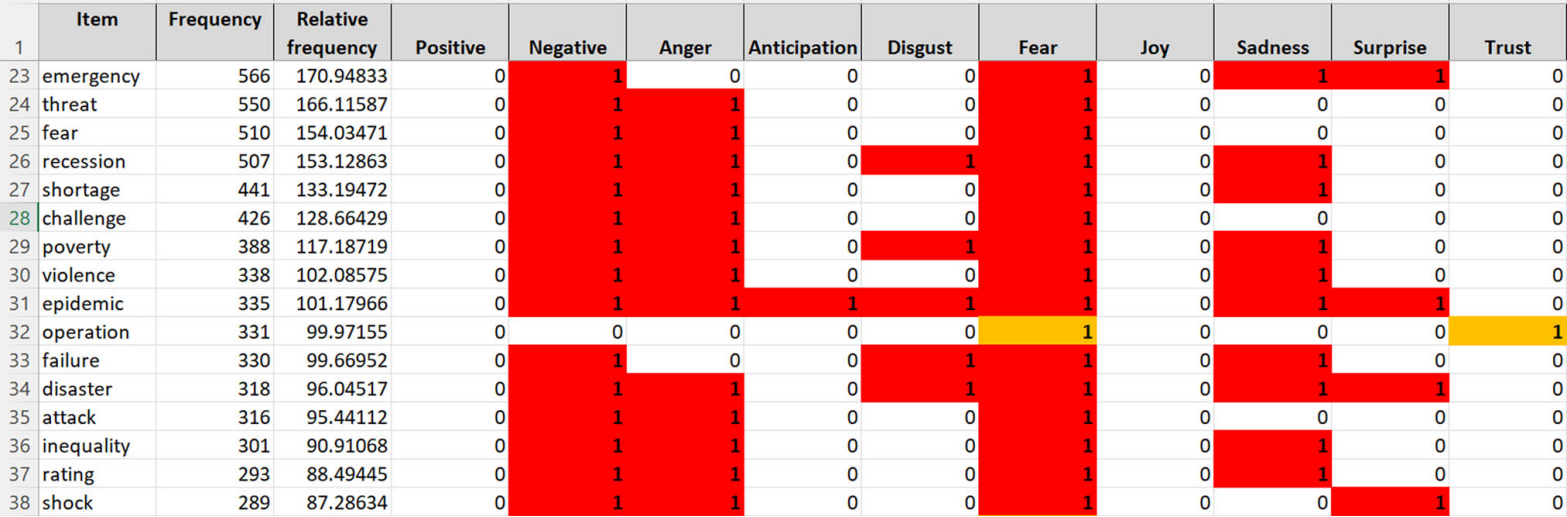
Sample of nouns associated with ANGER, FEAR, and other emotions in English.

With the word limit imposed by EmoLex, the result of the automatic search function is a list of unigrams by frequency with the polarity and emotions marked, as shown in Fig. 3, in which different colours have been assigned to make identification easier.

**Fig. 4:**
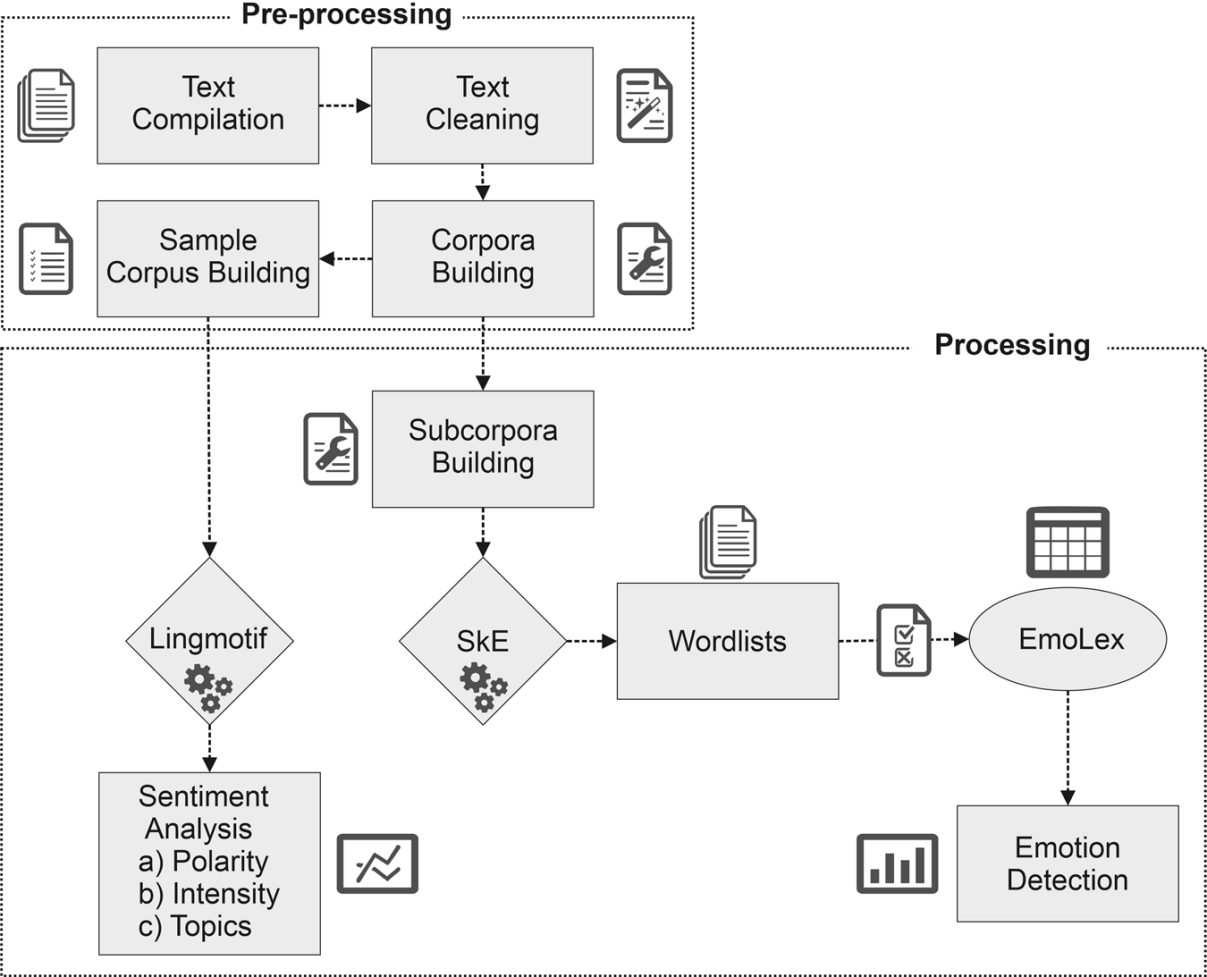
Methodological details and stages.

Figure 4 summarizes our methodological workflow, beginning with the compilation and cleaning of texts, followed by the building of the corpora, and ending with the processing of sub-corpora with the different tools and resources in order to perform sentiment analysis and emotion detection.

### Hypotheses and premise

In this study, we aim to explore the relationship between sentiment and emotion in financial journalism across languages and periods. Our investigation is grounded on two hypotheses and a guiding premise.

H_1_: There will be differences in sentiment polarity and intensity (understood as the proportion of positive or negative sentences versus neutral sentences) between the pre-COVID and COVID periods in each newspaper, becoming more negative in tone in the second period. In other words, no salient variations will be perceived between the EN and ES sub-corpora for the same period, both being similarly (if slightly) positive in the period before the crisis and significantly negative after the onset of the pandemic.

H_2_: The topics covered will change depending on the newspaper since their origins and scope are not the same, but the content will revolve mainly around critical financial matters in the first period but will focus on the havoc wrought by the global health crisis in the second period.

Premise: Given the linguistic variability of sentiment, we assume there will be notable variation in the ways the eight emotions appear in each sub-corpus, with even stronger differences between the two periods under analysis. The emotional lexicon should reveal different degrees of risk aversion and attraction according to our fear and greed scale.

By examining these hypotheses and premises, we aim to provide a comprehensive understanding of the role of sentiment and emotion in financial journalism across languages and time periods studied.

## Analysis and results

### Emotional polarity in the two periods studied

**Fig. 5:**
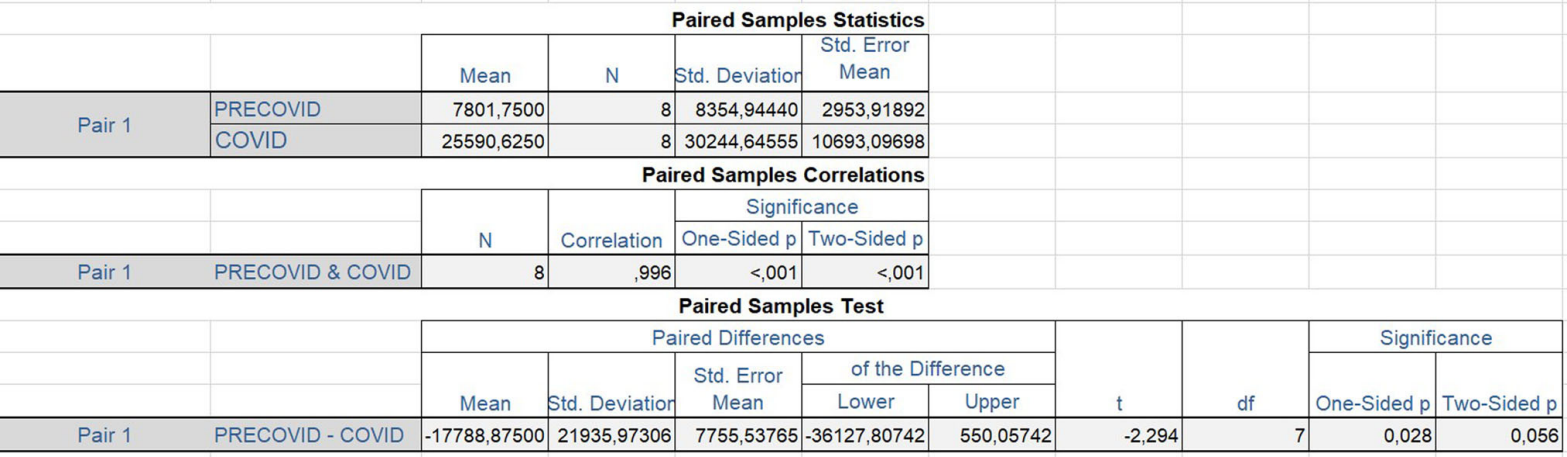
PRE-COVID EXPANSIÓN: polarity and intensity in sentences.

**Fig. 6:**
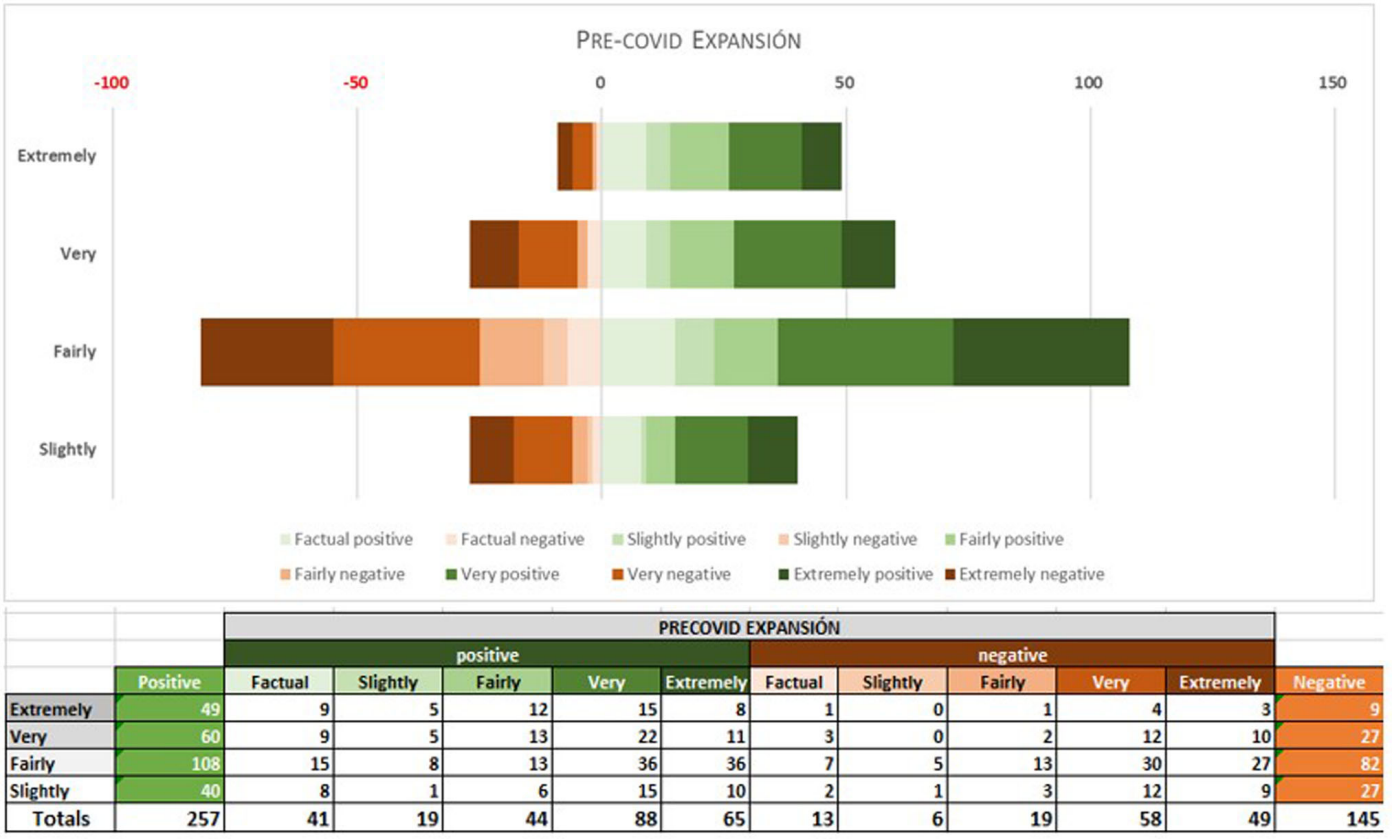
PRE-COVID ECONOMIST: polarity and intensity in sentences.

**Fig. 7:**
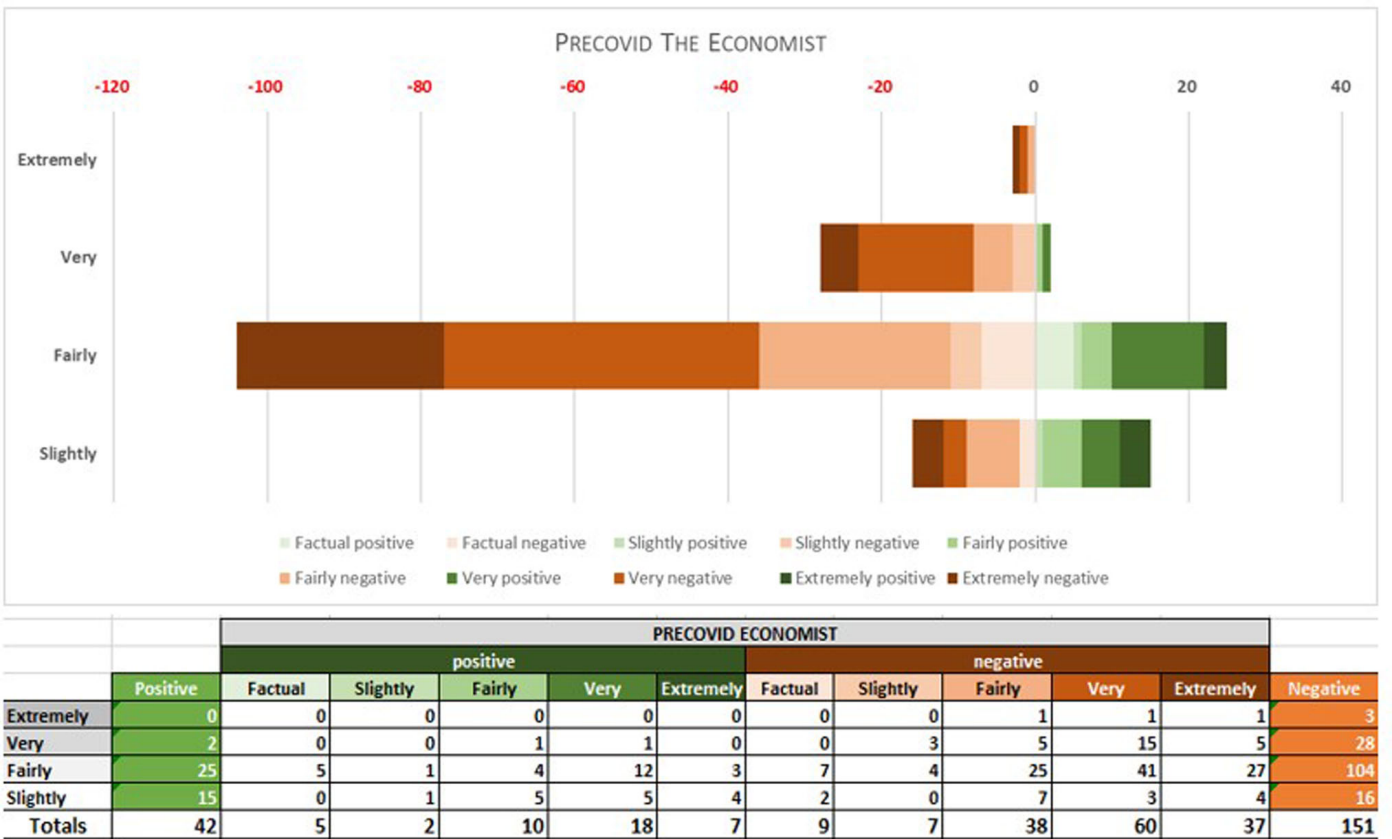
TSS and TSI averages in Precovid expansión and economist.

**Fig. 8:**
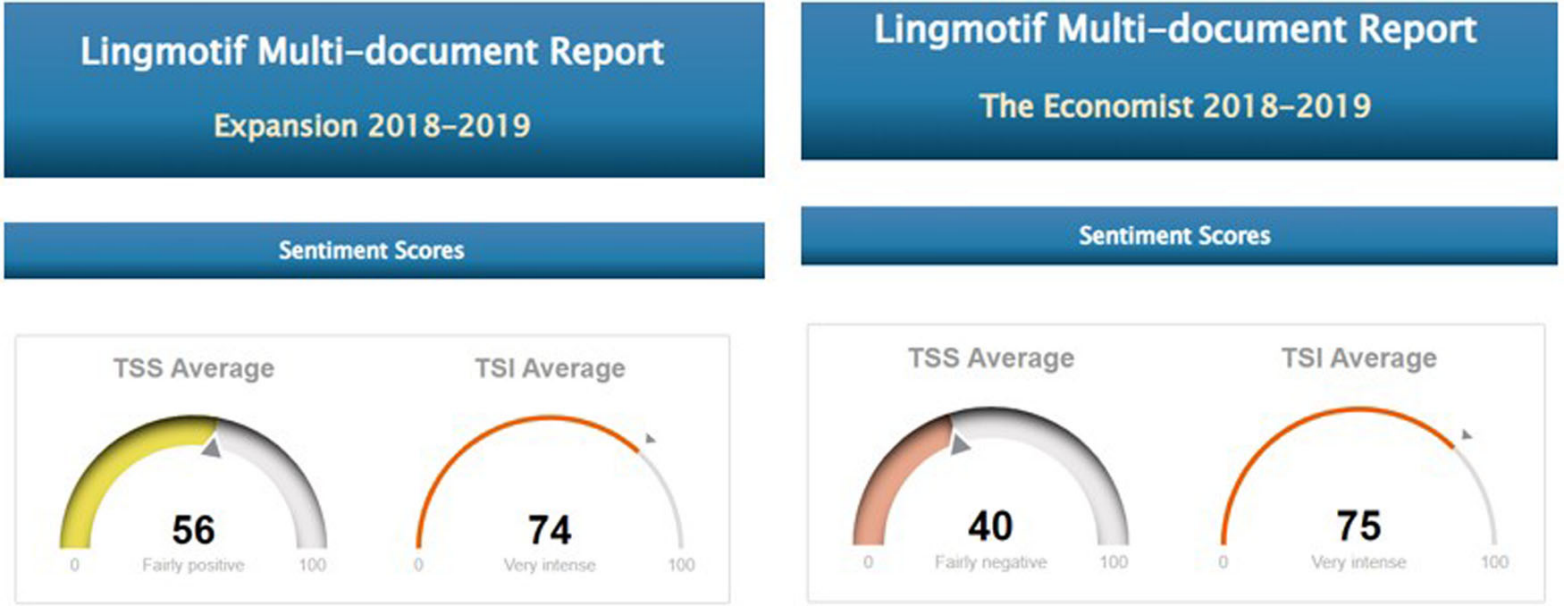
COVID EXPANSIÓN: polarity and intensity in sentences.

**Fig. 9:**
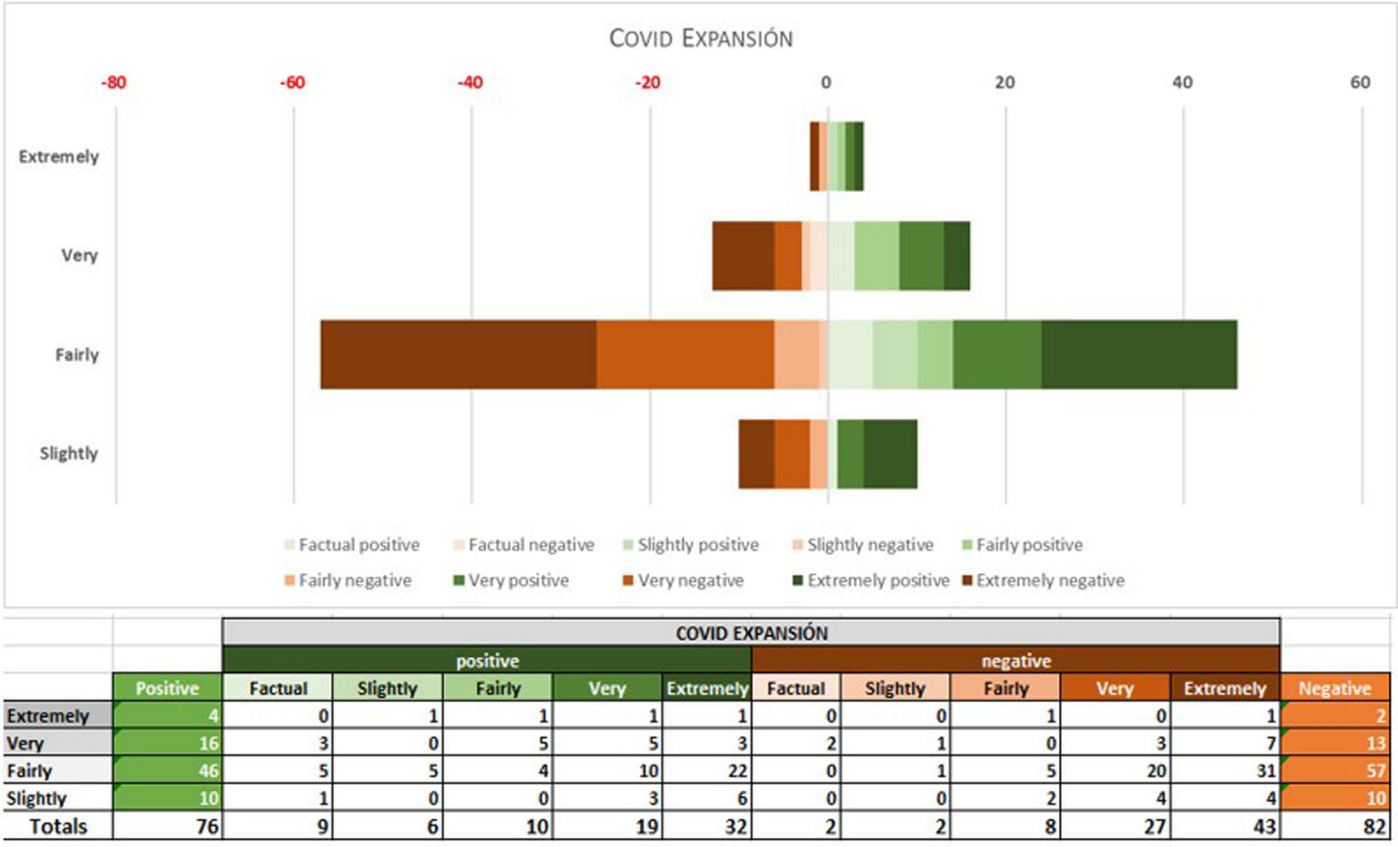
COVID ECONOMIST: polarity and intensity in sentences.

**Fig. 10:**
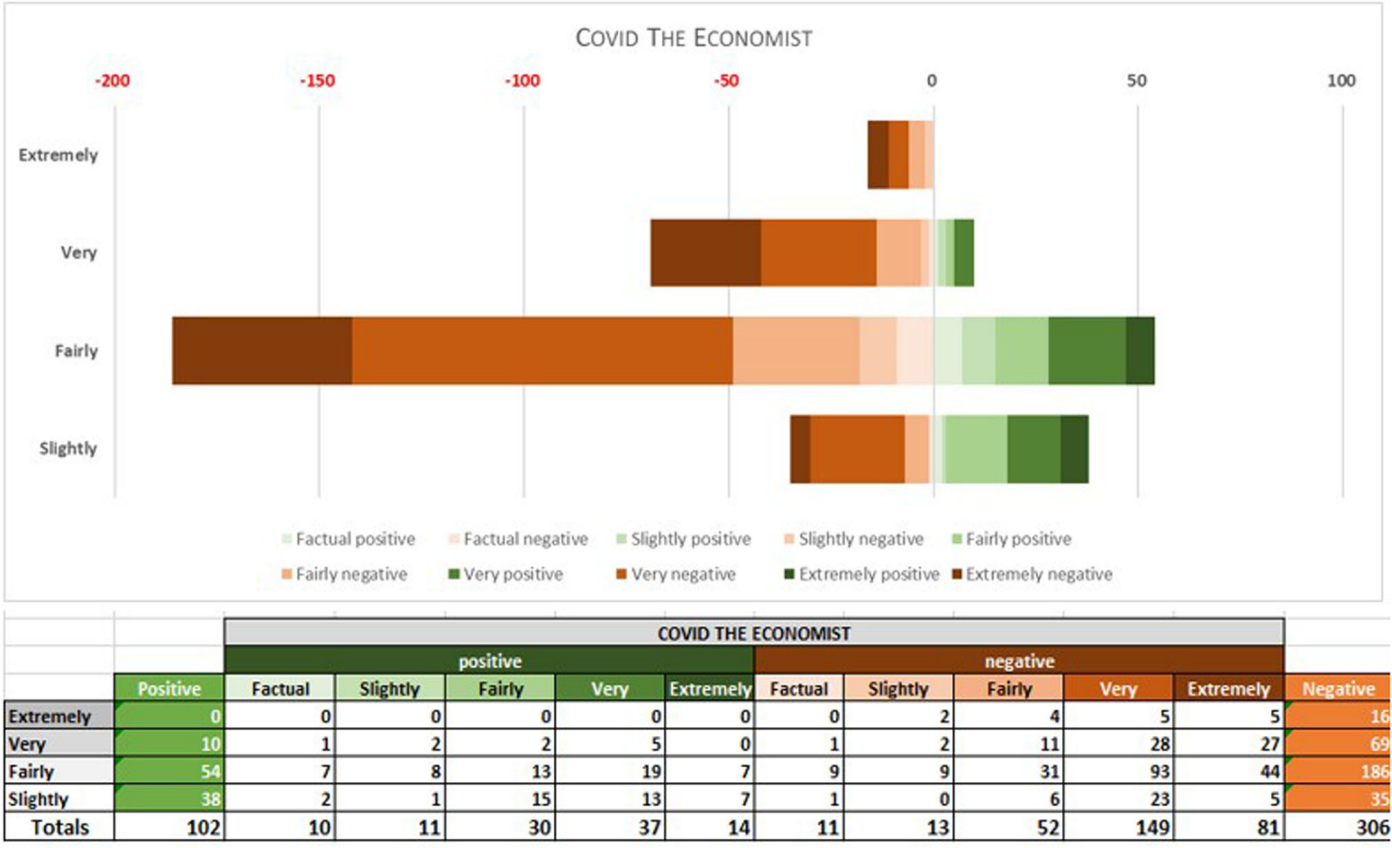
TSS and TSI averages in COVID EXPANSIÓN and ECONOMIST.

An initial analysis with a million-word sample per sub-corpus was made with Lingmotif 2, for the reasons explained above. The results shown in Figs. 5–10 correspond to the polarity and intensity of each sample from the pre-covid expansión, pre-covid economist, covid expansión and covid economist samples, respectively. As we can see, lexical items are rated as either positive/negative in terms of polarity (TSS) and as factual/slightly/fairly/very/extremely intense (TSI). Figures 5–7 show the results for the pre-COVID period in both periodicals.

As the graphs show, pre-covid expansión has 64% positive sentences (257 positive sentences), against 36% (or 145) negative ones (rating ‘fairly positive’ overall), TSI being ‘very intense’ (TSI average of 74). In pre-covid economist, by contrast, whereas the intensity values are similar to those of the Spanish periodical (TSI average of 75), polarity is ‘fairly negative’, with 42 positives (22%) compared to 151 negative items (78%), which indicates that the English newspaper is considerably less optimistic than the Spanish one.

Overall, the samples from the English periodical were rather more subdued in tone than those in Spanish with regard to economic expectations in the period before the pandemic, but emotional activity is almost identical in the two periodicals.

In the 2020–2021 period, however, the Spanish samples show a substantial reduction in positive items. Although negative and positive items have very similar values during this period, the sub-corpus as a whole tends marginally towards the negative, Lingmotif 2 classifying it as ‘slightly negative’ overall. The Spanish sub-corpus is as intense as the English one during this period (TSIs of 75 and 76, respectively), while the polarity values of the latter are rather more negative than in the pre-COVID period (with 306 negative items and 109 positive ones, indicating a significant increase in negativity, even though the software characterizes it as ‘fairly negative’ in terms of TSS).

Comparing results by periodical during the pandemic, the English sample shows a considerable increase in negative items in relation to the pre-COVID samples. In the Spanish case, the most notable decrease observed in the second period is that of positive words. Whereas in the pre-COVID period, 64% of the words were positive, during the COVID period there was a relative balance (76 positive vs. 82 negative words, 48% vs. 51%). It seems that the Spanish Newspaper *Expansión* does not want to create alarm among its readership, and this leads to the use of positive and negative lexis in roughly equal proportions. The English periodical is negative in both periods, as we have noted, but significant variations are seen between the pre-COVID and COVID periods, with a notable increase in negative (from 151 to 306) and positive (from 42 to 102) items in the second. It should be borne in mind that the emotional activity in both periodicals is ‘very intense’ in both periods.

A closer analysis of the corpus using Lingmotif 2 allows us to group the semantic character of positive and negative items around different lexical fields and their topic areas (see Table 4). The software also offers a list of the most frequent positive and negative items, the first ten of which are listed in Table 4.

As can be seen in Table 4, we organized the array of topics yielded by Lingmotif 2 into three large lexical fields for each publication and period. In the case of pre-covid expansión, the main issues of concern are climate change, business innovation, and the economy, the first of these being the most significant, as illustrated by the most frequent items, both negative and positive: *cambio climático* (‘climate change’, with 517 occurrences) and *sostenible* (‘sustainable’, 244), respectively. Other matters of interest include business innovation, that is, a (positive) change in business models (*mejorar*, ‘to improve’, with 176 occurrences, and *innovación*, ‘innovation’, with 106, for example) and (negative) economic affairs, with words like *riesgo* (‘risk’, 205), *crisis* (‘crisis’, 91 occurrences) and *deuda* (‘debt’, 77). Covid expansión, on the other hand, addresses these areas much less frequently, instead concentrating mainly on the health crisis, with strongly negative items such as *pandemia* (‘pandemic’, 887 occurrences), *contagio* (‘contagion’, 609) and *fallecido* (‘deceased’, 451). Some attention is also paid to political matters, but there is little reference to basic or directly economic issues. However, the number of topic areas addressed increases enormously in the English periodical, which also shows considerable uniformity in subject matter. Covid economist shows less lexical variability in terms of economic matters, but there is a clear abundance of words like ‘pandemic’ (the most frequent polarity item in the sub-corpus, with 940 occurrences), ‘virus’ and ‘lockdown’ (both with 350). Pre-covid economist shows an equally impressive array of macro- and micro-economic topics as its COVID equivalent but focuses mainly on negative issues such as the financial crisis and interest rates, using negative words such as ‘debt’ (206 occurrences) and ‘inflation’ (197). There is a surprising presence of the words ‘help’, ‘support’ and ‘rich’, which appear towards the top of the frequency lists of positive words in both pre-covid economist and covid economist, again illustrating the uniform editorial focus of this periodical.

## EmoLex and Plutchik’s emotions

**Fig. 11:**
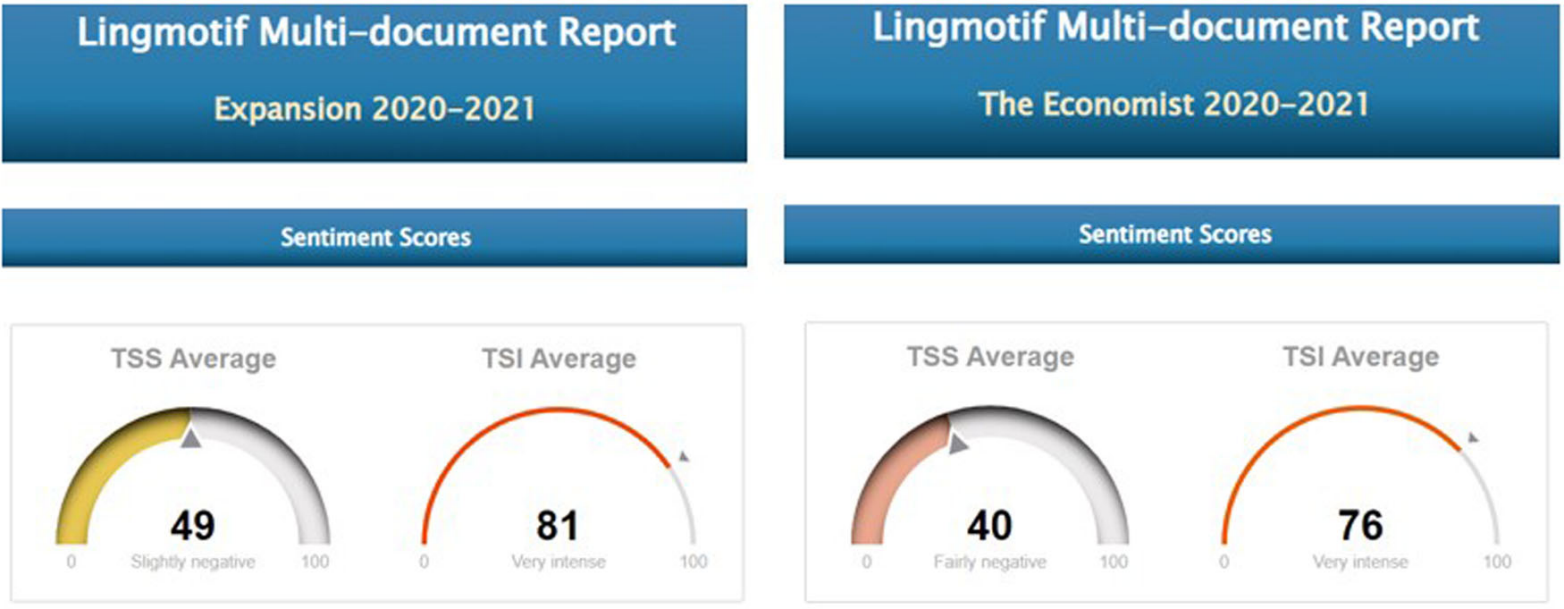
Positivity and negativity in the corpora, as percentages.

**Fig. 12:**
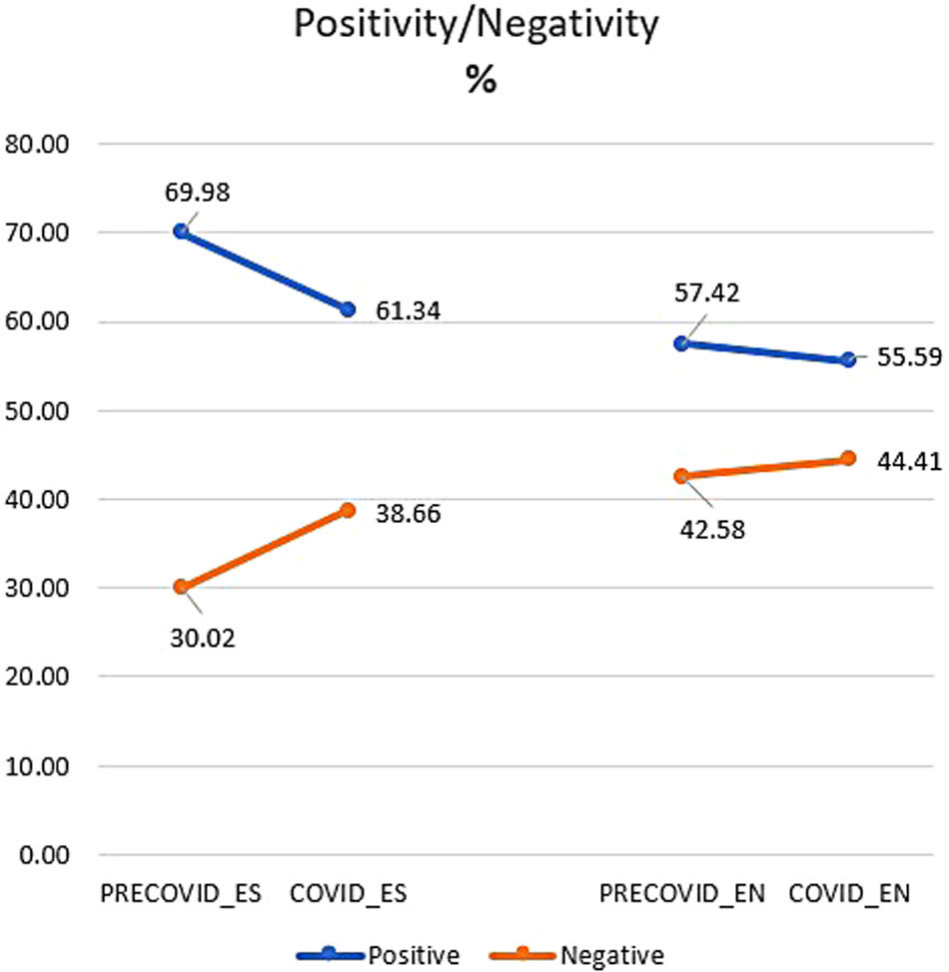
Emotions in PRE-COVID EXPANSIÓN and COVID EXPANSIÓN, as percentages.

**Fig. 13:**
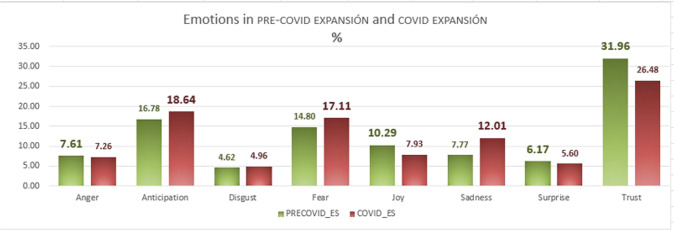
Emotions in PRE-COVID ECONOMIST and COVID ECONOMIST, as percentages.

**Fig. 14:**
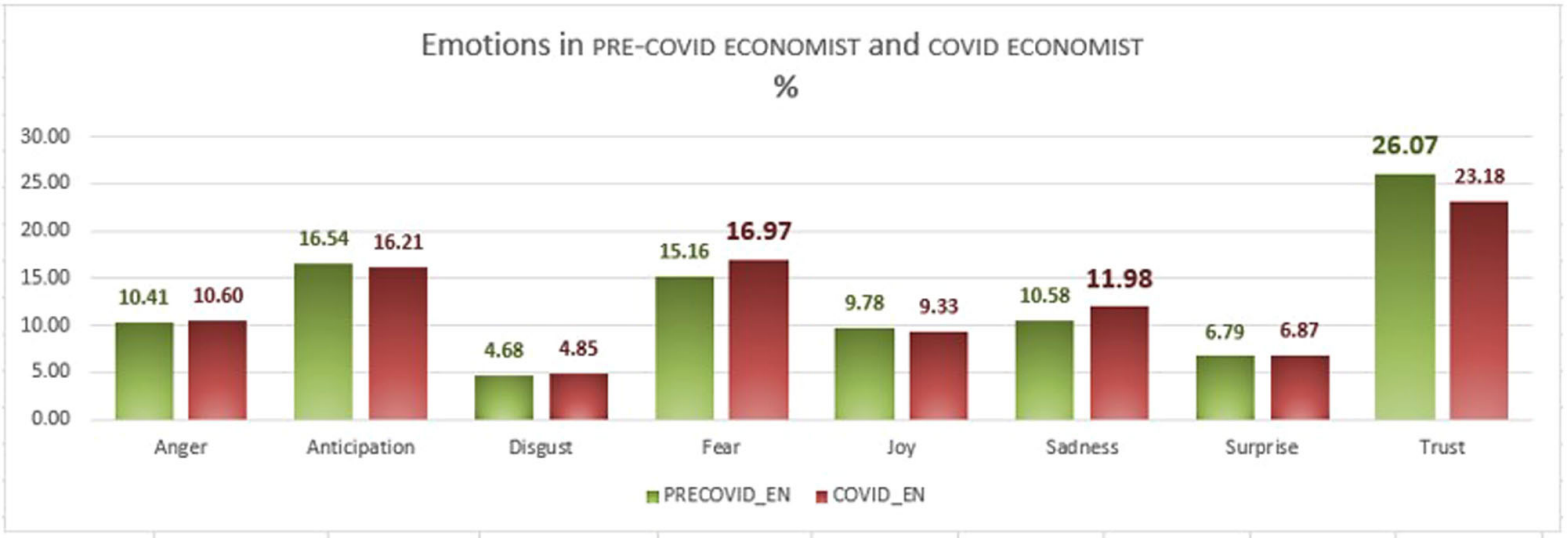
Variation of emotion values from pre-covid to covid, as percentages (*Expansión*).

**Fig. 15:**
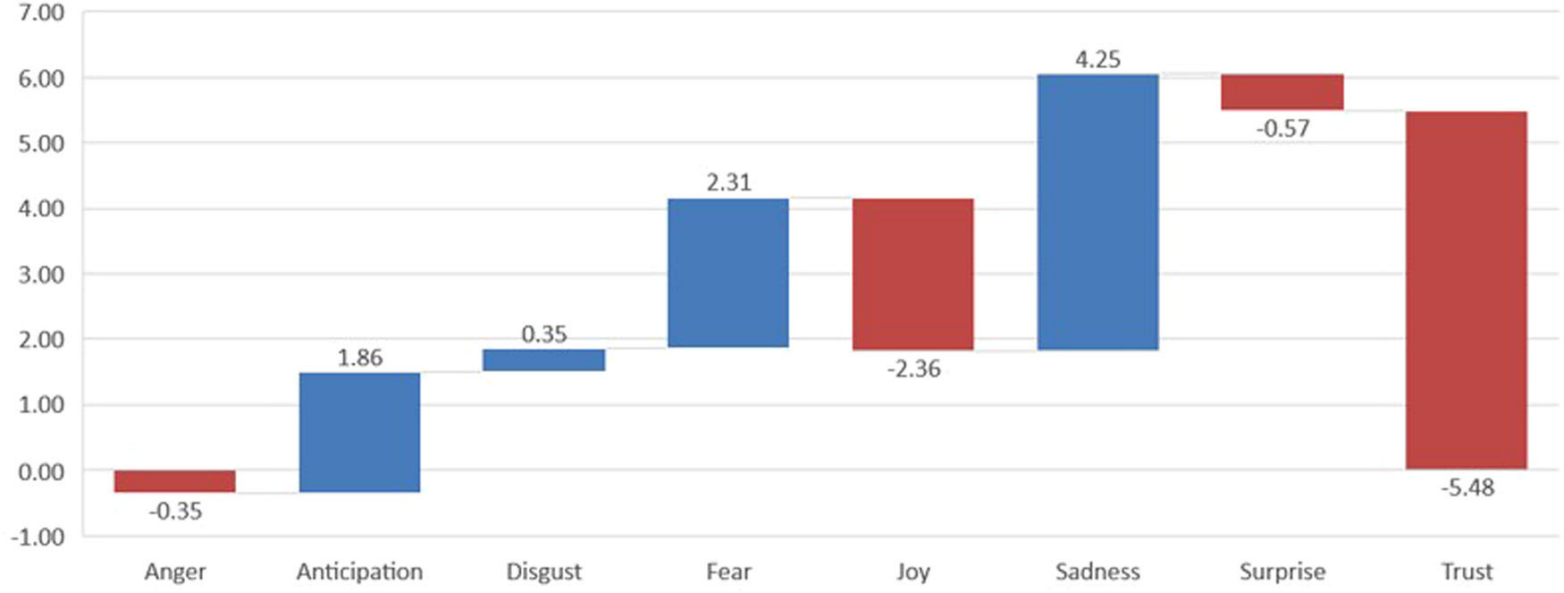
Variation of emotion values from pre-covid to covid, as percentages (*The Economist*).

Following our initial study, we conducted a more detailed examination of the data using EmoLex, itself based on Plutchick’s paradigm of eight basic emotions. For this we used the whole corpus, i.e., pre-covid expansión, pre-covid economist, covid expansión and covid economist (see Table 3). Figures 11–15 set out the results expressed as percentages, based on the relative frequency (the number of hits per million tokens) of each emotion. In order to know the relative frequency of each emotion, all the relative frequencies of words tagged with that specific emotion were tallied. The percentages were then obtained.

Figure 11 is very revealing, in that it confirms the results of the sample analysed with Lingmotif 2. Positive emotions substantially decrease between pre-covid- and covid expansión (69.98–61.34%), while negative ones increase (30.02–38.66%). Although declining positive and increasing negative trends are also identified in Economist, the differences are not as strong (57.42–55.59% for positive, 42.58–44.41% for negative). This suggests that the approach of the English periodical to news reporting is more stable than its Spanish counterpart.

Figures 12 (expansión) and 13 (economist) show the occurrence of the eight emotions in each corpus for each period.

If we look at the prevalent emotions in the expansión corpus, we see that the most notable of these in the first period are, in order: trust (31.96%), anticipation (16.68%) and joy, (10.29%). The negative emotions are relatively less frequent, for example, sadness (7.77%), fear (14.80%) and disgust (4.62%), while anger (7.61%)—in which the value is ambivalent, though mostly negative (more than 90% of the time) and seldom interpreted as positive by EmoLex—and the negative surprise (6.17%), both have intermediate values. Figure 12 illustrates that there are differences between periods, although once again trust has the highest value, despite dropping to 26.48%, and the incidence of joy also falls (7.93%). fear and sadness both increase in the covid sample (17.11% and 12.01%, respectively), as does anticipation (18.64%), while anger, disgust and surprise remain at the lowest values.

Turning to the economist data, these are shown in Fig. 13.

One of the evident issues arising from the analysis of this corpus is that the frequencies of emotions are similar in number to those in the Spanish corpus. Trust is again the most frequent, although it decreases in the second period (from 26.07 to 23.18%), while fear is the second most frequent emotion, although, by contrast, it increases in the second period, from 15.16 to 16.97%. Sadness also increases (from 10.58 to 11.98%). Anticipation is also an important emotion in the context of our material, yet contrary to the Spanish corpus, it decreases slightly in the second period (16.54–16.21%), as does joy (9.78–9.33%). Less dominant emotions are surprise and disgust, which show almost no change between periods. Figures 14 and 15 show the changes in values when we compare the two periods in the Spanish and English periodicals, respectively. The columns in red represent decreasing trends taking place in the periods; the blue columns represent increasing trends.

The emotions which have undergone the most variation from one period to the other are easily identified in the above graphs. Thus, the emotion that increased most in the Spanish pre-covid expansión to covid periods is sadness, followed by fear; that which decreases the most is trust. It is also interesting to compare the expansión and economist corpora here, in that the data are also striking in their differences; trust decreases substantially in both corpora, while sadness and fear increase; as with all the other emotions, though, the degree of variation is much less in both economist sub-corpora. Coincidences in the greater or lesser expression of emotions in the two periodicals are notable since it provides evidence that the economic atmosphere is similar in the narratives of both periodicals in both periods.

**Fig. 16:**
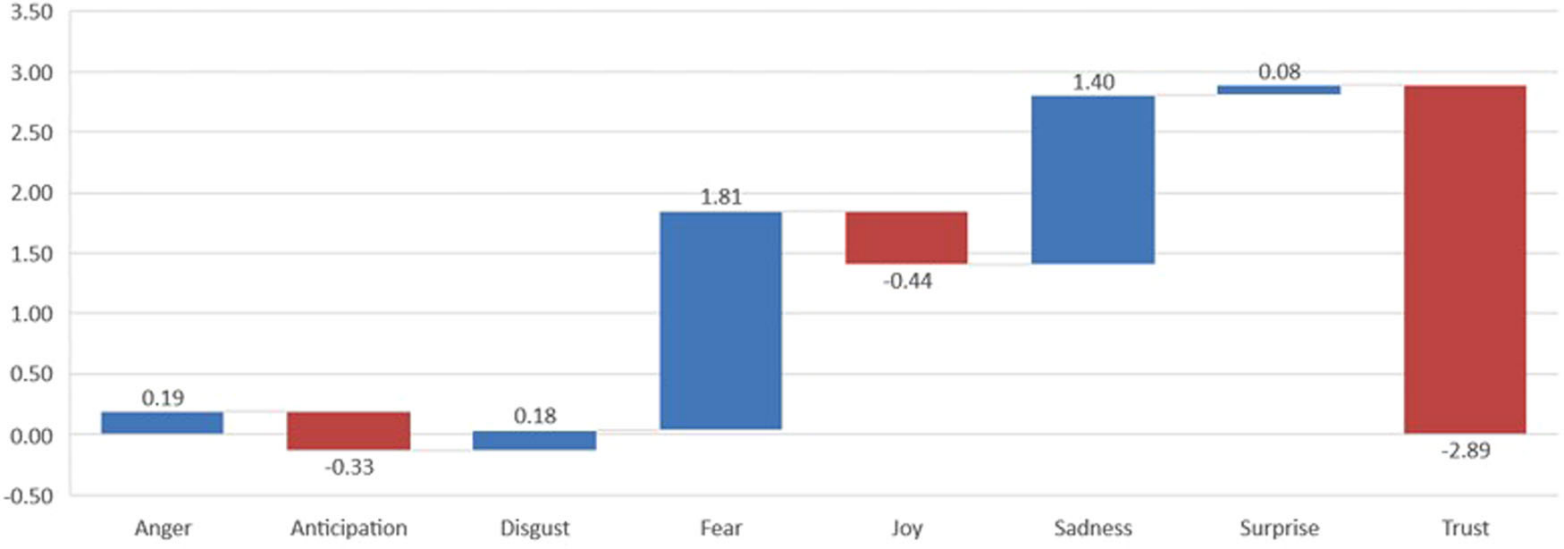
FEAR-GREED (ANGER+JOY) tendencies in the pre-COVID period.

The second stage of our analysis sought to focus specifically on the fear and greed taxonomy. Accordingly, we studied the 10 most frequent nouns exclusively relating to those two tendencies, some presented below along with their absolute frequency in brackets. Nouns were chosen because they represent the most frequently occurring word class in both corpora. This may be because specialized language is highly nominalized (Sager et al., 1980, p. 234), fulfilling as it does a mainly referential function. In what follows we will illustrate the most repeated nominal unigrams relating to the emotions that affect investor sentiment the most, i.e., those that EmoLex recognizes as relating to greed (anger+joy), and those relating to investor passivity or reticence, as represented by the emotional category of fear. Figure 16 presents the pre-covid data for both periodicals.

Interestingly, the data reveal that reticence to invest was higher in the Spanish sub-corpus (54%) prior to the pandemic. By contrast, greed was higher than fear (53% against 47%) in the English periodical during the same period. While it is true that we are only focusing on the extremes of the scale, we believe that this constitutes a useful means of exemplifying the e-implicatures that occur in nouns in regard to these extremes. Hence, words relating to fear in pre-covid expansión include *cambio* (‘change’, 2859.28^8^)*, gobierno* (‘government’, 1907.09)*, riesgo* (‘risk’, 725.29)*, lucha* (‘fight’, 495.68), *pérdida* (‘lost’, 195.84), *amenaza* (‘treat’, 159.37), *guerra* (‘war’, 149.92), while those relating to anger+joy include *reto* (‘challenge’, 436.25), *desafío* (‘challenge’, 284.98), *dinero* (‘money’, 151.27), *confianza* (‘trust’, 232.30), *mejora* (‘improvement’, 175.58). In pre-covid economist, the most frequent words representing a stimulus towards investing include ‘money’ (735.30), ‘share’ (690.62), ‘income’ (371.05), ‘demand’ (362.30), ‘cash’ (304.02), ‘wealth’ (189.41), ‘progress’ (146.67), ‘confidence’ (124.33), and ‘success’ (124.33), while those representing reticence to do so are ‘government’ (2259.33), ‘inflation’ (914.03), ‘change’ (610), ‘risk’ (582.80), ‘problem’ (505.10) ‘war’ (461.38), ‘recession’ (313.74), ‘threat’ (219.52), ‘loss’ (186.50), and ‘fear’ (165.13). Some examples from 2018, taken from both periodicals, are provided below.

1. Los eventos climáticos extremos, los desastres naturales y la falta de adaptación al cambio climático aparecen por primera vez entre los cinco principales riesgos globales (…)^9^.
  (*Extreme weather events, natural disasters, and failure to adapt to climate change appear for the first time among the top five global risks*).
2. En cuanto al futuro de estas energías en España, el regulador expresó su confianza en el mercado, que debe dar cabida a la generación renovable (…).
  (*Regarding the future of these types of energy in Spain, the regulator expressed its confidence in the market, which should be able to accommodate the generation of renewable energy*).
3. With wealth and success has come new confidence in a Chinese model and ambitions to share it.
4. When the next recession hits, as it eventually will, central banks may not have the leeway to cut short-term rates by the four percentage points or so that are the typical response to a downturn.

**Fig. 17:**
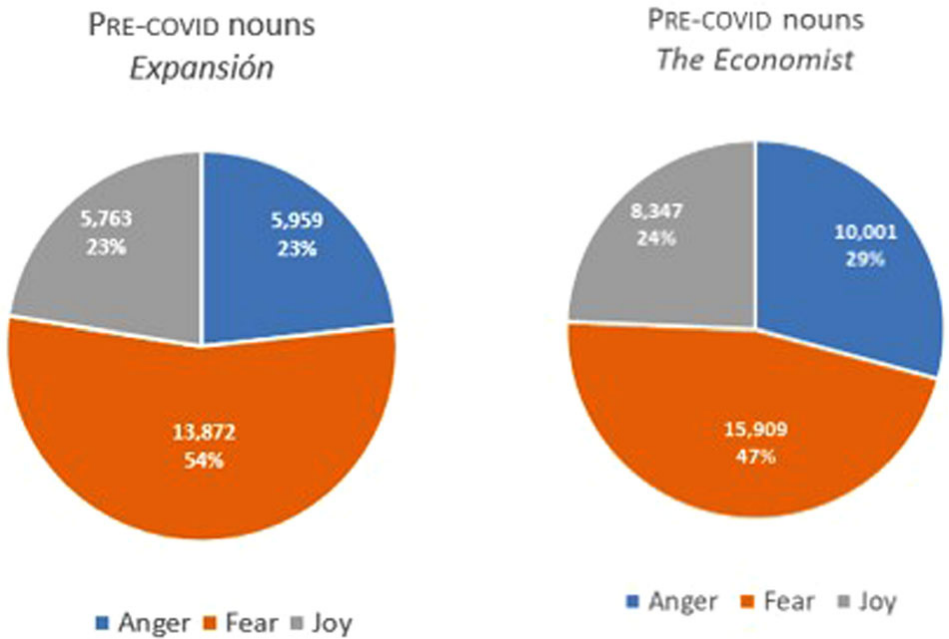
FEAR-GREED (ANGER+JOY) tendencies in the COVID period.

Figure 17 shows the same data with respect to the COVID period.

As we can see in Fig. 15, there is an increasing overall tendency towards fear in both periodicals compared to the period before the pandemic. The COVID period in both sub-corpora manifest fear through words relating to the health crisis, such as ‘pandemic’ (1463.63), ‘case’ (671.41)—with modifiers such as ‘covid-19’, ‘daily’, ‘severe’, ‘coronavirus’, ‘asymptomatic’—‘lockdown’ (495.32), ‘death’ (420.42), ‘disease’ (337.06) and ‘war’ (328.60) in the English sub-corpus. The Spanish counterpart for the same emotion is very similar, with words like *caso* (‘case’, 2096.27), *pandemia* (‘pandemic’, 1785), *contagio* (‘infection’, 883.21), *riesgo* (‘risk’ 551.44) *muerte* (‘death’, 411.82) and *confinamiento* (‘lockdown’, 197.90). The most frequent words relating to anger+joy in covid economist are ‘share’ (566), ‘money’ (506.8), ‘demand’ (353.97), ‘deal’ (315.02), ‘cash’ (300.82). In covid expansión, the most frequent words representing investing stimulus are *demanda* (‘demand’, 332.41), *ganador* (‘winner’, 286.93), *lucha* (‘fight’, 217.76), *reto* (‘challenge’, 232.49), *desafío* (‘challenge’, 146.03), *dinero* (‘money’, 110.8), *esfuerzo* (‘effort’, 142.83), *mejora* (‘improvement’, 99.27), and *libertad* (‘freedom’, 110.8). Below are some examples of this period (2020–2021).

1. Para contener la pandemia, Urkullu ha decretado el cierre de la hostelería y limitado el horario comercial de establecimientos de otros sectores, con la idea de no tener que recurrir al confinamiento domiciliario.
  (*To contain the pandemic, Urkullu has ordered by decree the closure of the hotel and catering industries and limited business hours for establishments in other sectors, with the idea of not having to resort to lockdown*).
2. La demanda eléctrica ha recuperado ya la mitad de lo perdido desde la declaración del estado de alarma.
  (*Electricity demand has already recovered half of what has been lost since the declaration of the state of emergency*).
3. But nationwide protests, the growing reality of the country’s economic turmoil and a rapidly spreading rebound of covid-19 cases have pushed him even farther behind.
4. Share prices may have rallied from the depths they plumbed when the coronavirus pandemic was spreading rapidly around the world.

### Hypothesis testing

The data used for hypothesis testing in this study was the frequency of negative polarity in news items in the newspapers under study before and after the declaration of the COVID pandemic. The data include the frequency values of adjectives, adverbs, nouns, and verbs in both languages. A paired sample *t*-test was selected in SPSS software to analyse the data.

The question posed to test the hypothesis was the following: What effect has COVID had on the positive and negative sentiment variables reflected in certain publications in two Spanish and English periodicals? The answer to this question is, according to our study, that there is a significant difference in the negative polarity before the declaration of the pandemic and after the declaration. Given this question and its answer, the baseline hypothesis and its null hypothesis were posed, the baseline hypothesis being the following:

H_1_: there are no significant differences in the means of negative sentiment expressed in the newspapers studied before and after the declaration of COVID as a pandemic.

Consequently, to test the hypothesis, we put forward the null hypothesis (H_0_), formulated as follows:

H_0_: there are no significant differences in the levels of negative sentiment expressed in selected newspapers before and after the declaration of COVID-19 as a pandemic.

We decided to use a paired sample *t*-test because of our two related groups of data (the pre-COVID and the COVID variables) for the following reasons:

1. Our study contains values collected before and after the onset of the pandemic, which makes it a longitudinal study.
2. We had two fixed variables: the pre-pandemic variable (2018–2019) and the post-pandemic variable (2020–2021).
3. The random variable being the number of occurrences of terms in our samples is a numerical variable, suitable for our test.

In hypothesis testing, *p*-value is a measure of the evidence against the null hypothesis. It represents the probability of obtaining a test statistic as extreme as, or more extreme than, the one observed, assuming the null hypothesis is true (Brezina, 2018). If the *p*-value is less than or equal to the significance level (*α*) chosen by the researcher (e.g., *α* = 0.05), then the null hypothesis is rejected in favour of the alternative hypothesis (H_1_). On the other hand, if the *p*-value is >*α*, then there is not enough evidence to reject the null hypothesis. If the probability obtained (i.e., *p*-value) is ≤*α*, being *α* = 0.05 in our case, we reject the null hypothesis (H_0_) and accept the alternative hypothesis (H_1_). Conversely, if the obtained probability (i.e., *p*-value) is >*α*, H_0_ is not rejected and H_1_ is not accepted.

The data we used to carry out the test correspond to the frequency values of negative polarity in the total of adjectives, adverbs, nouns and verbs in Spanish and English extracted from the pre-covid and covid corpus (Table 6).

**Table 6:** Variables used in Student’s *t*-test.

	NEG. Freq.	
	PRE-COVID	COVID
ADJ-EN	6339	22,212
ADV-EN	377	1342
NOUN-EN	24,885	90,152
V-EN	11,959	38,054
ADJ-ES	2853	6173
ADV-ES	43	79
NOUN-ES	12,309	38,229
V-ES	3649	8484

**Fig. 18:**
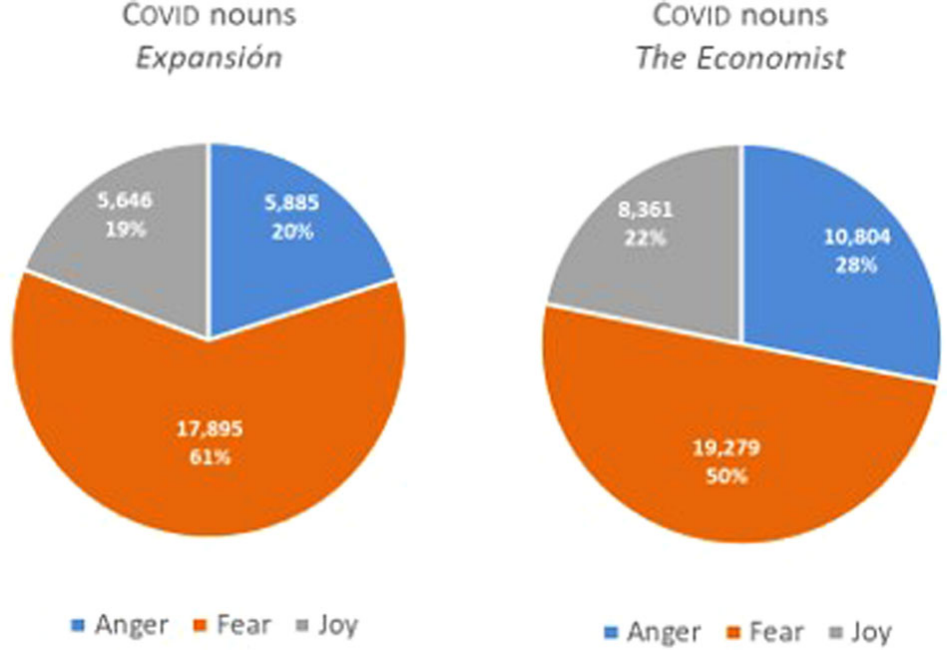
Paired samples test results for negative sentiment.

Figure 18 shows the paired sample *t*-test result obtained using SPSS software.

The results of the paired sample *t*-test indicate that there is a statistically significant difference between the mean negative frequencies before and after the pandemic, with a *p*-value of 0.028 (one-sided) or 0.056 (two-sided) at a significance level of 0.05. The mean difference between the negative frequencies before and after the pandemic is −17,788.875, with a standard deviation of 21,935.973 and a standard error of 7755.537. The 95% confidence interval of the difference ranges from −36,127.807 to 550.057.

The negative mean difference suggests that the negative frequency of the selected newspapers increased after the pandemic was evident. This finding is consistent with the increases in negative sentiment observed across all parts of speech in both *The Economist* and *Expansión*.

The strong correlation (*r* = 0.996) between the pre-pandemic and post-pandemic data pairs further supports the validity of the Student’s *t*-test results, indicating that the data is consistent and reliable.

For H_2_, we used a frequency list with a relative degree of co-occurrence frequency (DOCF) from Sketch Engine, as it allowed us to compare the relative frequency of different topics in each newspaper corpus and identify differences between the two periods. We then compared the relative frequency of topics related to critical financial matters and the global health crisis in each newspaper corpus in the first and second periods, respectively.

We studied nouns, as they often represent concrete or abstract concepts, entities, or ideas, which makes them particularly useful for identifying the main topics and themes within a corpus. Nouns often provide a more stable and consistent representation of topics and tend to be more specific and less ambiguous than other parts of speech, such as adjectives or verbs. We selected those nouns which had a DOCF higher than 20% in pre-COVID and 25% in COVID periods to focus on the topics that appear in a substantial proportion of the documents, ensuring that our analysis captured the main subjects that are frequently discussed across the corpus and making the analysis more manageable and easier to interpret^10^.

Based on the frequent words from the *Expansión* newspaper corpus during the years 2018 and 2019, it seems that the articles cover a wide range of topics.

For the 2018–2019 period, the summary is as follows:

1. The main topics in this period seem to be related to politics, economy, and business, with terms like *gobierno*, *presidente*, *empresa*, *economía*, and *país* having high frequencies and DOCFs.
2. There is a notable focus on European issues, as indicated by the presence of words like *euro* and *Europa*.
3. Environmental and sustainability topics are present, as shown by terms like *energía*, *sostenibilidad*, and *cambio climático*.

For the 2020–2021 period, the summary is:

1. The COVID-19 pandemic becomes a dominant topic, as evidenced by the high frequencies and DOCFs of words like *pandemia*, ‘covid-19’, ‘covid’, and ‘coronavirus’.
2. There is still a strong focus on politics, economy, and business, with terms like *gobierno*, *país*, *empresa*, *sector*, *España*, and *economía* being frequently mentioned.
3. The importance of international politics, investments, and development is visible through the presence of words like *Europa*, ‘China’, *fondo*, *inversión*, and *tecnología*.
4. Environmental and sustainability issues continue to be covered, as indicated by terms like *transición*, *energía*, *emisión*, *lucha*^11^, and *sostenibilidad*.

Concerning these two periods in *Expansión* newspaper, we can conclude that the distribution of documents by topic shows that politics, economy, and business were the primary topics in both periods. The COVID-19 pandemic emerged as a dominant topic in the 2020–2021 period, reflecting its global impact. Environmental and sustainability issues were present in both periods, although not as dominant as other topics.

The data in *The Economist* were, for the period 2018–2019:

1. Strong focus on economic and financial topics, including macroeconomic issues, monetary policy, and financial markets. Examples of related words include ‘rate’, ‘interest’, ‘bank’, ‘market’, ‘economy’, ‘growth’, ‘price’, ‘investor’, ‘inflation’, ‘trade’, ‘job’, ‘tax’, ‘currency’, ‘dollar’, and ‘loan’.
2. Frequent coverage of political events, leadership, and policy issues, with a focus on specific countries and regions such as America, China, and Europe. Examples of related words include ‘country’, ‘government’, ‘president’, ‘minister’, ‘election’, ‘party’, ‘law’, ‘reform’, ‘leader’, ‘union’, ‘politician’, ‘budget’, ‘European’, and ‘security’.
3. Coverage of social issues and demographic topics, highlighting the newspaper’s interest in reporting on various aspects of society and the impact of economic and political developments on people’s lives. Examples of related words include ‘people’, ‘university’, ‘worker’, ‘home’, ‘job’, ‘income’, and ‘city’.

And for the period 2020–2021:

1. Increased coverage of health-related topics due to the global health crisis, with a focus on pandemic-related issues such as lockdowns, viruses, and deaths. Examples of related words include ‘pandemic’, ‘covid-19’, ‘lockdown’, ‘virus’, and ‘death’.
2. Continued coverage of politics and government, with a focus on global affairs and geopolitical matters. Examples of related words include ‘government’, ‘president’, ‘state’, ‘policy’, ‘minister’, ‘party’, ‘election’, ‘law’, ‘rule’, and ‘order’.
3. Continued coverage of economic and financial topics, including analysis of economic trends and business developments, as well as finance and investment topics. Examples of related words include ‘economy’, ‘firm’, ‘business’, ‘market’, ‘company’, ‘rate’, ‘bank’, ‘crisis’, ‘job’, and ‘growth’.
4. Analysis of social concerns related to the pandemic, such as the impact on workers, cities, and schools. Examples of related words include ‘people’, ‘health’, ‘worker’, ‘city’, ‘population’, ‘family’, and ‘school’.
5. Continued focus on financial markets and investment strategies. Examples of related words include ‘share’, ‘price’, ‘interest’, ‘capital’, ‘investment’, ‘investor’, ‘spending’, and ‘deal’.

The frequency of economic and financial topics is consistently high in both periods in this *The Economist*, but there is a clear shift in focus in the 2020–2021 period due to the global health crisis. This shift is evident in the increased coverage of health-related topics and the analysis of social concerns related to the pandemic. At the same time, there is a continued emphasis on political and government issues, with a focus on global affairs and geopolitical matters. The changes in the topics covered suggest that the origins and scope of the newspaper have a significant influence on its content, but overall, the content revolves around critical financial matters in the first period and the havoc wrought by the global health crisis in the second period.

It can be concluded that H_2_ is supported by the previous analysis of both newspapers, as they both reflect a shift in focus towards the impact of the global health crisis on different aspects of the economy and society.

## Discussion

This study was formulated on three hypotheses, all of which have been confirmed to a greater or lesser degree.

First, we predicted that there would be differences between the pre-COVID and COVID periods in terms of intensity and sentiment, with both periodicals being similarly positive (although only slightly) in the period prior to the crisis, and considerably negative after the pandemic began. This hypothesis has not been fully supported, since the four sub-corpora have proved to be similarly intense in the high levels of emotional activity recorded, thus our initial assumption that economic reports are highly charged in emotional terms is not confirmed. Regarding polarity, it is true that both the Spanish and the English samples show a notable increase in negative items, this being especially so in the Spanish case, where a decrease in the number of positive items in the second period is also evident, as demonstrated by our initial study with Lingmotif 2, and subsequently confirmed in the analysis using EmoLex. The English data do not confirm our initial expectations either, since results are almost equally negative in both periods, albeit with slight variations which were more noticeable in the initial study than in the subsequent, overall analysis using the emotion lexicon.

Second, we predicted that the range of topics would change according to periodicals, in tune with the nature of each publication, and this was indeed confirmed through the close analysis of topics covered using Lingmotif 2 and DOCF in Sketch Engine for topic distribution. In our prediction, it was implicit that the subject matter in the pre-COVID period would be less sombre in tone than in the COVID period. This was seen to be true to a certain extent, in that the variation here is only very slight in the case of the English periodical. We predicted that the subject matter of the first period would revolve around economics and business, while the second period would focus on the COVID crisis, and this we assumed would be the case for both publications. But in fact, this is not entirely so, at least for the English newspaper. *Expansión* does focus on the economy in the first period, but in the second it focuses almost all its attention on the pandemic. By contrast, the range of economic and business topics covered is much broader in *The Economist*, both before and during the pandemic, confirming the more rounded and comprehensive nature of this publication. While it deals with a wide range of economic issues, it is also true that the main concern of the periodical in the second period is the pandemic and its effects, yet within these concerns, the interest rate crisis and inflation are realities that are mentioned in *The Economist*, whereas they are entirely absent from *Expansión*.

Our third hypothesis was that there would be clear variations in the way that the eight emotions were present, both in each sub-corpus and between sub-corpora, with even greater differences between the two periods. This would indicate diverse degrees of risk aversion and attraction, on the basis of our adaptation of the fear and greed scale of the financial markets. However, the typology of the most frequent emotions is in fact quite similar in both corpora, with trust in general being the most frequently occurring emotion in the periodicals, though it decreased in the second period, no doubt reflecting the turmoil of the pandemic. Regarding the FEAR-GREED scale that regulates behaviour in terms of investing or selling, the presence of TRUST suggests a moderate tendency towards attraction to risk, which in both periodicals decreases and is replaced by a growing sense of risk aversion in the second period, which is also characterized by the greater presence of SADNESS, another of the most common emotions in the two corpora, which also strongly rejects risk-taking.

Other emotions are less commonly found, but ANGER and JOY (both basic emotions in Plutchik’s scale)—which taken together embody the intense emotion GREED, itself understood as competition for resources and goal achievement—are represented in greater proportions than the other emotions, but once again the reduction in their use in the second period suggests that the drive to succeed in terms of attaining and possessing also decreases in both corpora from the first to the second period. All in all, we find that both periodicals show a global tendency toward moderate risk-taking, which is greatly ameliorated by the presence of FEAR in the second period. Given that the two periodicals under investigation here are both very prominent in their respective spheres of influence, it seems probable that their dissemination would have had consequences in terms of the behaviour of investors in general.

The second part of our study, involving the use of EmoLex for a finer analysis of the data in search of the words that best express the tendencies of attraction/aversion in the financial atmosphere, confirmed all the above claims. A sample was taken of the ten most frequent words representing the main emotions greed (anger+joy) and fear in both periodicals and both periods, and the trend towards retraction in investment in the more recent sub-corpora was confirmed: although greed was more common in the English publication than in the Spanish one in both periods, its decline in favour of fear was notable in both periodicals in the second period, with nouns providing clear evidence of the effects of the health crisis, this tendency adding to a latent one in the pre-COVID period that underlined risks relating to climate change, inflation, and the trade conflicts of the time.

## Conclusion

The present study has explored the connection between sentiment and economic crises, as verbalized through the use of emotional words in two periodicals. In doing so, we have considered the ways in which the economic crisis, both immediately prior to and during the pandemic, was conveyed by two highly specialized newspapers, one in English, the other in Spanish, and how events influenced emotional expression and the language used to this end in the two periods. We have confirmed that emotional polarity was moderately negative to mildly positive in both *Expansión* and *The Economist*, although the former maintained a more optimistic tone prior to the pandemic. This indicates that, in the pre-COVID period, with the effects of the financial crisis still biting, the texts published in the English periodical conveyed the belief that some grounds for optimism existed and that the reactivation and recovery of the economy would begin to appear on the horizon. Turning to emotional expression during the pandemic, we saw how both newspapers painted a gloomy picture of the economic situation, and while the outlook as detected in the Spanish corpus deteriorates relatively more acutely, the English periodical maintains a more measured, although still somewhat bleak, tone in both periods.

To ascertain in greater detail how this expression of emotion would affect activity in the financial markets, we designed a scale based on Plutchik’s eight-emotion paradigm, which we applied to the CNN Stock Market Index (Fear & Greed). In theory, the Fear and Greed Index acts as a barometer for whether the stock market is fairly priced by looking at the emotions of investors. We know that market behaviour can be affected by emotions that transmit risk attraction or aversion and that the verbalization of these sentiments by such prestigious newspapers carries considerable weight in terms of investor outlook and behaviour. If this is so—and we have effectively found a way to connect mathematical algorithms (such as those used by stock markets) with emotion words—then it is probably possible to demonstrate that economics is indeed more intimately connected to feelings than purely rational economic theories would have us believe.

## Data Availability

The datasets generated during and/or analysed during the current study are available from the corresponding author on reasonable request.

## Footnotes

1 As analysed by the IMF (https://cutt.ly/FLoIIiN) and the OECD (https://cutt.ly/ELoIX5U).

2 https://www.imf.org/en/Publications/WEO.

3 https://www.imf.org/en/Publications/WEO.

4 CNN Money Fear and Greed indicators: https://money.cnn.com/data/fear-and-greed/.

5 In Sketch Engine, the term “token” is used to refer to the basic units of text that are processed by the system. Tokens can be individual words (tokens which begin with a letter of the alphabet) but they can also be punctuation marks or other elements of the text (nonwords). In our analysis words will be the focus of study, as they carry the meaning in a text and are the units that are used to form sentences and convey ideas.

6 This multi-language text analytics suite was developed by the Tecnolengua group at the University of Malaga and constitutes a more sophisticated and comprehensive evolution of Lingmotif 1, the original SA software developed by the group.

7 EmoLex versions in other languages were created by translating the English terms using Google Translate in August 2022 (https://saifmohammad.com/WebPages/NRC-Emotion-Lexicon.htm).

8 Relative frequency, i.e. number of hits per million tokens.

9 For all the Spanish examples we provide our own translation for a better understanding.

10 The whole wordlists of each subcorpora generated during the current study and showing the frequency, relative frequency, DOCF and relative DOCF are available in the form of a spreadsheet at t.ly/cD5a6.

11 *Lucha* (‘fight’) can also be applied to pandemic topic, but concordances showed it was more frequent “lucha contra el cambio climático” (rel. freq. 179.86) than “lucha contra el coronavirus|pandemia|covid” (rel. freq. 13.9).
